# Skeletal Muscle Quality Assessment in Patients With Cardiac Disease Associated With Type 2 Diabetes Mellitus: A Pilot Study

**DOI:** 10.7759/cureus.51897

**Published:** 2024-01-08

**Authors:** Momo Takahashi, Yuma Tamura, Tomoki Tsurumi, Masato Terashima, Harunori Takahashi, Hajime Tamiya, Tomoki Furuya, Yuki Nakatani, Naoyuki Otani, Takanori Yasu

**Affiliations:** 1 Department of Rehabilitation, Dokkyo Medical University Nikko Medical Center, Nikko, JPN; 2 Department of Physical Therapy, Niigata University of Health and Welfare, Niigata, JPN; 3 Department of Cardiovascular Medicine and Nephrology, Dokkyo Medical University Nikko Medical Center, Nikko, JPN; 4 Department of Diabetes and Endocrinology, Dokkyo Medical University Nikko Medical Center, Nikko, JPN; 5 Department of Cardiology, Dokkyo Medical University Nikko Medical Center, Nikko, JPN

**Keywords:** weight-bearing index, cardiac rehabilitation, peak oxygen uptake, type 2 diabetes mellitus, skeletal muscle quality, echo intensity, phase angle

## Abstract

Background

Type 2 diabetes mellitus (T2DM) is associated with changes in skeletal muscle quantity and quality, such as increased ectopic fat. Cardiac rehabilitation (CR) aims to improve the exercise capacity and muscle strength. This study aimed to determine the relationship between qualitative changes in the skeletal muscles and exercise function in patients with and without diabetes mellitus.

Methods

The study included patients with cardiovascular diseases who entered CR. Of 72 CR patients (68.1±9.0 years) who underwent a cardiopulmonary exercise test and skeletal muscle assessment at discharge, 15 patients with T2DM and 15 without DM were selected using propensity score matching by age and gender.

Results

No significant differences in the skeletal muscle echo intensity (EI) (T2DM: 58.4, Non-DM: 53.4, p=0.32), skeletal muscle index (T2DM: 7.5 kg/m^2^, Non-DM: 7.2 kg/m^2^, p=0.36), or the weight-bearing index (WBI)(T2DM: 0.44, Non-DM: 0.50, p=0.35) existed between the two groups. The phase angle (PhA) (T2DM: 3.67°, Non-DM: 4.49°, p<0.05) and peak oxygen uptake (T2DM: 12.3 mL/kg/min, Non-DM: 14.8 mL/kg/min, p<0.05) were significantly lower in the T2DM group. PhA values showed a significant correlation with the WBI, a parameter of lower limb muscle strength (r=0.50, p<0.05).

Conclusion

The coexistence of cardiovascular disease and T2DM resulted in a decrease in the PhA, indicating a qualitative decrease in skeletal muscle mass. The PhA is also associated with lower limb muscle strength.

## Introduction

According to the 10th edition of the International Diabetes Federation Diabetes Atlas, the global diabetes population in 2021 will continue to grow to 537 million people or one in every 10 adults [1]. Heart failure (HF) is one of the most common complications of diabetes mellitus, and type 2 diabetes mellitus (T2DM) is an independent risk factor for HF [2]. Worldwide, 30 million patients with HF have reached 30 million [3]. The co-occurrence of T2DM and HF worsens the prognosis of HF and contributes to all-cause and cardiovascular mortality [4]. In addition, we recently reported that in patients with T2DM and established coronary artery disease (n=7,785), an incremental association between the baseline white blood cell count as an inflammatory marker and the primary cardiovascular outcome of HF events requiring hospitalization in a retrospective cohort [5]. An important prognostic factor for patients with heart disease is exercise capacity, such as peak VO_2 _[6]. Improving muscle strength and exercise tolerance with exercise therapy is important for secondary prevention of cardiovascular events [7].

The skeletal muscles of patients with T2DM are characterized by increased intramuscular ectopic fat, which increases insulin resistance [8,9]. In contrast, exercise therapy decreases intramuscular fat mass [10]. The qualitative improvement of skeletal muscles is particularly important for preventing severe diseases in patients with T2DM. Qualitative assessments of muscles have been reported using magnetic resonance imaging, proton magnetic resonance spectroscopy, and X-ray computed tomography [11]. As a simpler method, skeletal muscle echo intensity (EI) using ultrasound measurement devices is also used for qualitative assessment of skeletal muscle. Reviews have reported that EI correlates with age [12], sex [13], and intramuscular fat [14]. Furthermore, the EI of the rectus femoris muscle in chronic heart failure (CHF) is significantly higher than in healthy individuals [15]. The EI is also used as a simple qualitative assessment of skeletal muscle [16] and a prognostic and nutritional indicator for various diseases [17,18]. The phase angle (PhA) decreases with the severity of HF in both men and women [19], and the relationship between the PhA and survival rate in patients with HF has been reported to show that the survival rate decreases as the PhA decreases [20].

Although skeletal muscle changes in T2DM patients with cardiovascular disease have been observed in scattered cases [21], there have been limited reports on qualitative changes, and the effects on motor functions, such as muscle strength and exercise tolerance, are unknown in patients with T2DM and established cardiovascular disease. This pilot study aimed to compare the qualitative assessments of skeletal muscle and physical function in cardiac rehabilitation (CR) patients with T2DM and without T2DM as a pilot study.

## Materials and methods

This was a single-center retrospective study. The study protocol was approved by the Ethics Committee of Dokkyo Medical University Nikko Medical Center (ethical license number Nikko 22-009). This study was conducted following the “Declaration of Helsinki” by the World Medical Association and the “Ethical Guidelines for Medical and Health Research Involving Human Subjects” by the Ministry of Education, Culture, Sports, Science, and Technology and the Ministry of Health, Labor, and Welfare (established on December 22, 2014, and partially revised on March 27, 2023). Informed consent was obtained from all the participants.

**Figure 1:**
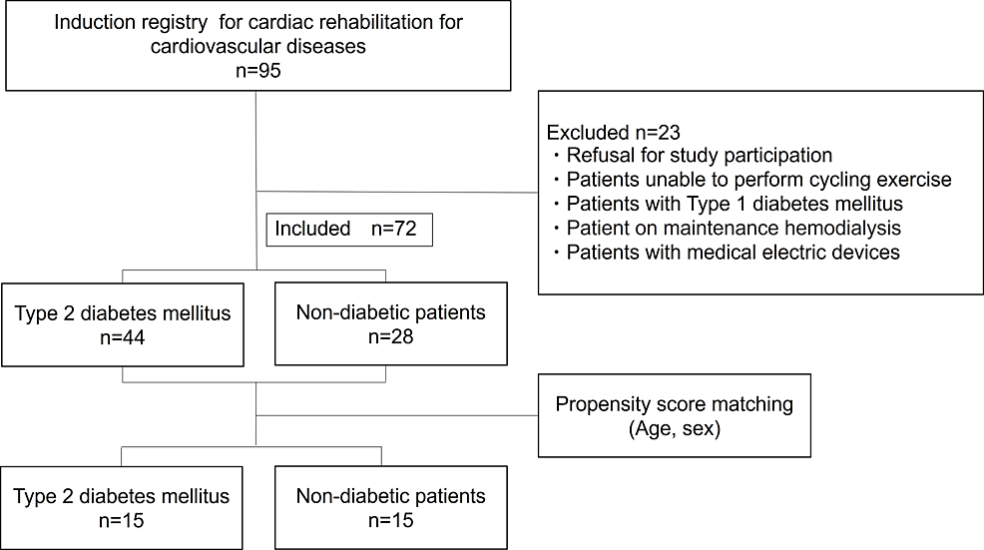
Flow chart of patient selection and propensity score This was a single-center, retrospective study. The participants included 72 patients with cardiovascular disease, 44 with type 2 diabetes, and 28 without diabetes. A propensity score was determined based on age and sex, with 15 patients classified as having type 2 diabetes and 15 as non-diabetic.

Seventy-two patients with cardiovascular disease were admitted to Dokkyo Medical University Nikko Medical Center and underwent cardiac rehabilitation (CR). They registered in the Cardiac Rehabilitation Database from June 2015 to February 2017 and underwent a cardiopulmonary exercise stress test and skeletal muscle evaluation at discharge or the start of outpatient care. Patients admitted with a diagnosis of T2DM underwent drug therapy within one month of evaluation. They were enrolled in the T2DM group, and those admitted without a diagnosis of T2DM were included in the Non-DM group. Both groups were adjusted for age and sex by propensity score matching (Figure 1).

Bioelectrical impedance analysis (BIA)

The participant stood barefoot on the measurement table, held the handle in a standing position for approximately 15 seconds, and the measurement was taken. The Skeletal Muscle Index (SMI), which was calculated by summing the amount of skeletal muscle in the limbs/height m^2^, and body fat percentage (body fat kg/body weight kg × 100), was calculated using a multi-frequency body composition analyzer (TANITA, MC-180) [22]. The PhA was calculated using the formula 'Phase angle= arctangent (reactance/resistance) × 180°/π'. Measurements were taken from the right leg.

Skeletal muscle echo intensity (EI)

Ultrasound images of the right leg RF muscle were obtained using a B-mode ultrasound measurement device (EnVisor, Philips, Amsterdam) [12]. The measurement position was at the midpoint between the anterior superior iliac spine and the lateral epicondyle of the femur. The ultrasound gain, frequency, and scanning depth were measured as 50, 11, and 6 cm, respectively. Imaging was repeated three times, and the median values of quadriceps muscle thickness and subcutaneous fat thickness were used as the measured values. EI was calculated using ImageJ image analysis software (National Institutes of Health, Bethesda, MD, USA; Laboratory for Optical and Computational Instrumentation [LOCI], University of Wisconsin-Madison, Madison, WI, USA). Muscle luminance in the ROI was indicated as an arbitrary unit (AU) on a scale of 0 (black) to 255 (white). The reliability of EI has been demonstrated in a previous study [10]. We confirmed the inter-rater reliability of the EI measurement of the rectus femoris muscle according to a previously established protocol [12].

Body composition measurements and exercise capacity

The weight-bearing index (WBI) was calculated by measuring the isometric knee extension muscle strength (kgf) of the right lower extremity using a pull sensor for manual muscle testing (SAKAIMED, MT-150) and dividing by body weight (kg) [23].

Peak VO_2_, VE/VCO_2_ slope, and peak RER were measured using an ergometer for exercise testing (BK-ERG-121, Mitsubishi, Japan), and expiratory gas analysis was performed using the ramp loading method with an aeromonitor (AE310, Minato Medical Science, Japan) [24]. Peak VO_2_ was defined as VO_2_ attained during peak exercise to the point of symptom-limited maximal work. The VE/VCO_2_ slope was calculated by linear regression analysis and calculated below the respiratory compensation point.

Statistical analyses

Data for continuous variables were expressed as mean ± standard deviation based on the normality of the distribution assessed using the Shapiro-Wilk test. Categorical variables were expressed as numbers (n) and percentages, and the χ^2^ test was used for between-group comparisons. IBM SPSS Statistics for Windows, Version 27 (Released 2020; IBM Corp., Armonk, New York, United States) was used for statistical analyses. Statistical significance was set at a two-sided p-value <5%. Differences in motor function between patients with T2DM and those without DM complications were examined using Student's t-test, and the relationship between muscle quality assessment and other items was examined using Pearson's product rate correlation coefficient.

## Results

Patients’ baseline characteristics

Table 1 presents the baseline characteristics (age, sex, BMI, and laboratory data) of each group before and after propensity score matching. There were no significant differences between the groups after age and sex matching, with 15 patients in each group. Comorbidity was significantly different for hypertension and CKD. There were also no significant differences in laboratory data.

**Table 1:** Patients’ baseline characteristics Data are shown as % or mean ± SD. CRP, C-reactive protein; Hb, hemoglobin; HDL-C, high-density lipoprotein cholesterol; LDL-C, low-density lipoprotein cholesterol; T2DM, type 2 diabetes mellitus. p-values were obtained using Student's t-test; p*:  p<0.05.

	Before Matching		After Matching		p-value after matching
	Non-DM	T2DM	Non-DM	T2DM	
	n=44	n=28	n=15	n=15	
Age (years)	69.7±10.2	70.3±8.6	68.1±9.4	68.1±8.9	1.00
Male, n(%)	29 (66)	20 (71)	9 (60)	10 (67)	0.72
Body mass index (kg/m^2^)	23.4±5.2	24.2±4.8	25.7±4.3	24.3±4.6	0.41
Hypertension, n(%)	34(79.0)	22(75.9)	8(53.3)	13(86.7)	0.049*
Dyslipidemia, n(%)	31(72.0)	21(72.4)	9(60.0)	10(66.7)	0.72
Chronic kidney disease, n(%)	12(27.9)	16(55.2)	3(20.0)	11(73.3)	0.002*
Current smoker, n(%)	24(55.8)	18(62.0)	7(46.7)	6(40.0)	0.15
Atrial fibrillation, n(%)	11(25.6)	3(10.3)	4(26.7)	1(0.07)	0.16
Hb (g/dl)	13.0±1.7	12.7±1.5	13.9±1.3	13.2±1.8	0.25
HbA1c (%)	6.2±0.9	7.0±2.2	6.0±0.2	7.1±2.1	0.17
White blood cells (10^2^/μl)	5.60±1.58	6.42±1.88	6.18±1.85	6.85±2.44	0.41
HDL-C (mg/dl)	45.9±12.7	43.9±8.1	45.0±12.0	42.5±8.8	0.62
LDL-C (mg/dl)	89.4±37.8	74.0±15.1	82.7±30.0	74.7±14.5	0.46
Triglyceride (mg/dL)	135.9±79.3	123.1±69.4	147.1±88.5	101.0±38.8	0.08
Creatinine (mg/dL)	1.06±0.65	1.20±1.48	1.00±0.23	0.97±0.45	0.84
eGFR (ml/min/1.73m^2^)	56.6±20.0	62.6±16.4	58.0±20.7	59.8±15.8	0.81
CRP(mg/dl)	0.26±0.29	0.19±0.25	0.15±0.12	0.12±0.18	0.65

Table 2 shows the cardiovascular diseases leading to hospitalization. The underlying diseases were not significantly different between groups (p=0.23).

**Table 2:** Crosstabulation of DM and Non-DM AMI, acute myocardial infarction; AP, angina pectoris; CHF, chronic heart failure; LEAD, lower extremity arterial disease; T2DM, type 2 diabetes mellitus.

		AMI	AP	LEAD	CHF (AHA/ACC class c)	Previous hospitalization for HF	Other diseases	p-value
T2DM	n (%)	6 (40.0)	4 (26.7)	0 (0)	4 (26.4)	11	1 (6.7)	0.23
Non-DM	n (%)	3 (20.0)	2 (13.3)	1 (6.7)	9 (60.0)	7	0 (0)	
All	n (%)	9 (30.0)	6 (20.0)	1 (3.3)	13 (43.3)	18	1 (3.3)	-

The medications used are listed in Table 3. Non-DM were not taking any diabetes medications. Diabetes medications in T2DM were most commonly biguanides. Antihypertensive medications were most commonly beta-blockers in both groups. T2DM's diabetes history was 5.7±3.2 years.

**Table 3:** Medication Data are shown as % or mean ± SD.  ARB, angiotensin receptor blocker; ACEi, angiotensin-converting enzyme inhibitor; type 2 diabetes mellitus.

	Non-DM n=15	T2DM n=15
			Insulin, n (%)	0 (0)	0 (0)
Glucagon-like peptide-1 receptor agonist, n (%)	0 (0)	4 (26.7)
Biguanide, n (%)	0 (0)	12 (80.0)
Dipeptidyl peptidase-4 inhibitor, n (%)	0 (0)	10 (66.7)
Sodium glucose cotransporter 2 inhibitor, n (%)	0 (0)	3 (20.0)
Thiazolidinediones, n (%)	0 (0)	1 (6.7)
Sulfonylurea drug, n (%)	0 (0)	1 (6.8)
Glinide, n (%)	0 (0)	0 (0)
Alpha-glucosidase inhibitor, n (%)	0 (0)	0 (0)
ARB/ ACEi, n (%)	10 (66.7)	6 (40.0)
Beta blocker, n (%)	11 (73.3)	9 (60.0)
Ca channel blocker, n (%)	9 (60.0)	9 (60.0)
Diuretics, n (%)	8 (53.3)	6 (40.0)
Angiotensin receptor-neprilysin inhibitor, n (%)	0 (0)	0 (0)
Mineral corticoid-receptor antagonists, n (%)	2 (13.3)	2 (13.3)
Anti-platelet, n (%)	8 (53.3)	11 (73.3)
Direct oral anticoagulant, n (%)	2 (13.3)	1 (0.07)
Warfarin, n (%)	2 (13.3)	2 (13.3)
History of diabetes (years)	0	5.7±3.2

Body composition measurements and exercise capacity

Table 4 compares body composition measurements and exercise capacity assessments, including BIA, ultrasonography, WBI, and peak VO_2_. There were no significant differences in SMI and muscle thickness, muscle brightness, and WBI. In contrast, the PhA and peak VO_2_ were significantly lower in T2DM.

**Table 4:** Body composition measurements and exercise capacity Data are shown as % or mean ± SD. DM, Diabetes mellites; T2DM, type 2 diabetes mellitus. p-values were obtained using Student's t-test; p*:  p<0.05.

	Non-DM	T2DM	p-value
				Body fat percentage (%)	26.7±8.8	28.3±12.7	0.85
Skeletal muscle mass (kg)	43.8±6.3	41.7±7.5	0.59
Skeletal muscle index (kg/m^2^)	7.2±2.3	7.5±1.4	0.36
Phase angle (°)	4.49±0.89	3.67±0.58	0.007*
Rectus femoris echo intensity (AU)	53.4±14.3	58.5±12.1	0.32
Femoral anterior subcutaneous fat thickness (cm)	0.99±0.41	0.92±0.45	0.64
Quadriceps femoris muscle thickness (cm)	2.88±0.75	2.70±0.71	0.51
Weight-bearing index (kg/kg)	0.50±0.19	0.44±0.11	0.35
Peak oxygen uptake (ml/kg/min)	14.8±3.6	12.3±2.1	0.038*

Relationship between the phase angle and outcome

**Figure 2:**
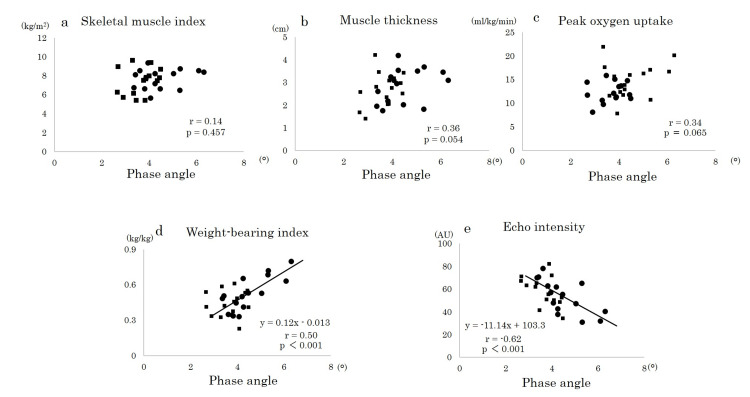
Relationship between the phase angle and other outcomes (n=30). The figures show the correlation between the phase angle and other parameters such as skeletal muscle index (a), quadriceps femoris muscle thickness (b), peak oxygen uptake (c), weight-bearing index (d), and echo intensity (e) in both groups. A significant correlation was found between the phase angle and weight-bearing index and between the phase angle and echo intensity. A Pearson’s correlation analysis was performed.   Square (■) indicates T2DM and circle (●) indicates Non-DM.

There were no significant correlations between the PhA and SMI (Figure 2a), quadriceps femoris muscle thickness (Figure 2b), and peak VO_2_ (Figure 2c). On the other hand, there were significant correlations between the PhA and WBI (Figure 2d, r=0.50, p<0.001), EI (Figure 2e, r=-0.62, p<0.001).

## Discussion

In CR patients with T2DM, PhA and peak VO_2_ were significantly lower than those without T2DM, even under matched background conditions. Furthermore, the PhA positively correlated with WBI. The present study provides useful information for evaluating skeletal muscle disorders in patients with CR, with or without T2DM.

The present results are consistent with the previous reports showing that CR patients with concomitant DM had significantly lower peak VO_2_ than those without T2DM [25]. Nishihara et al. have reported that the concurrent group with T2DM and cardiovascular disease showed 10.3% lower peak VO_2_ than that of Non-DM cardiovascular disease patients [26]. Glycemic control, left ventricular dysfunction, and autonomic neuropathy have been cited as factors that cause further decline. In addition, a decrease in muscle quality due to increased intracellular lipid content in the skeletal muscles has been reported as a skeletal muscle disorder caused by T2DM [7]. Furthermore, muscle quality is considered an important indicator in skeletal muscle assessment because it changes prior to muscle mass loss [27]. Therefore, we evaluated muscle quality using the EI and PhA, which are simple and feasible muscle quality evaluation methods in the clinical setting. The present results showed that only PhA levels were significantly decreased in patients with T2DM compared to those without T2DM. Since a PhA less than 4.2° is a predictor of all-cause mortality in patients with cardiac disease [20], the PhA of T2DM was 3.62°, suggesting that T2DM may cause a further decline in skeletal muscle quality among cardiac diseases. EI is reportedly higher in patients with failure than in healthy subjects [12]. EI was slightly, but not significantly, higher in the DM group. Nishihara et al. (2021) have reported that in community-dwelling older Japanese adults with and without diabetes, 351 men (72.7 ± 6.9 years old) with and without DM show no significant difference in EI of the rectus femoris muscle [26]. Patients with heart disease complicated with T2DM may produce inflammatory cytokines, such as tumor necrosis factor-α and interleukin-6, due to intramuscular fat accumulation, which induces insulin resistance and leads to skeletal muscle catabolism [28]. Longstanding hyperglycemia is thought to promote mitochondrial dysfunction and loss of protein synthesis in the skeletal muscle of patients with T2DM [28]. Additionally, increased extracellular fluid volume due to edema, a symptom of heart failure, and the effects of the extracellular matrix may be related to changes in muscle quality [29].

PhA assessment is a simple and important tool for evaluating skeletal muscle quality and is associated with prognosis, especially in patients with T2DM and heart disease [30]. Muscle strength is the sum of several factors, including muscle mass and quality [31], and is more strongly associated with prognosis than muscle mass in CR [32]. The PhA correlated with muscle strength in the present study. Consequently, the PhA is an extremely important assessment tool for patients with CR.

Limitations of the study

First, this was a single-center, cross-sectional study with a small sample size; secondly, we did not clarify the relationship between muscle quality and exercise tolerance. In the future, we plan to increase the sample size and consider the effect of disease bias. A longitudinal study is also needed to examine the effect of the rehabilitation intervention.

## Conclusions

In this study, the PhA and exercise tolerance were significantly lower in CR patients with T2DM. The PhA in patients with T2DM and cardiovascular disease was below the cutoff value, a predictor of all-cause mortality, indicating poor muscle quality. Furthermore, the PhA correlated with WBI in the presence or absence of T2DM, suggesting that the PhA may be useful in the physical assessment of CR. Muscle quality is an important indicator of skeletal muscle assessment because it changes before muscle mass loss. A longitudinal study with a larger sample size is needed to examine the effects of rehabilitation.
